# Supplementary material for: A universal dimensionality function for the fractal dimensions of Laplacian growth
Source: Sci Rep. 2019 Feb 4;9:1120. doi: 10.1038/s41598-018-38084-3 (PMC6362037; doi:10.1038/s41598-018-38084-3)
Supplement: Supplementary file 1 — Supplementary Figure S1 [file 41598_2018_38084_MOESM1_ESM.pdf]

Supplementary Information to "A universal dimensionality function for the fractal dimensions of Laplacian growth"

.

J. R. Nicolás-Carlock* and J. L. Carrillo-Estrada

Instituto de Física, Benemérita Universidad Autónoma de Puebla, Apdo. Postal. J-48, Puebla 72570, México.

*jnicolas@ifuap.buap.mx

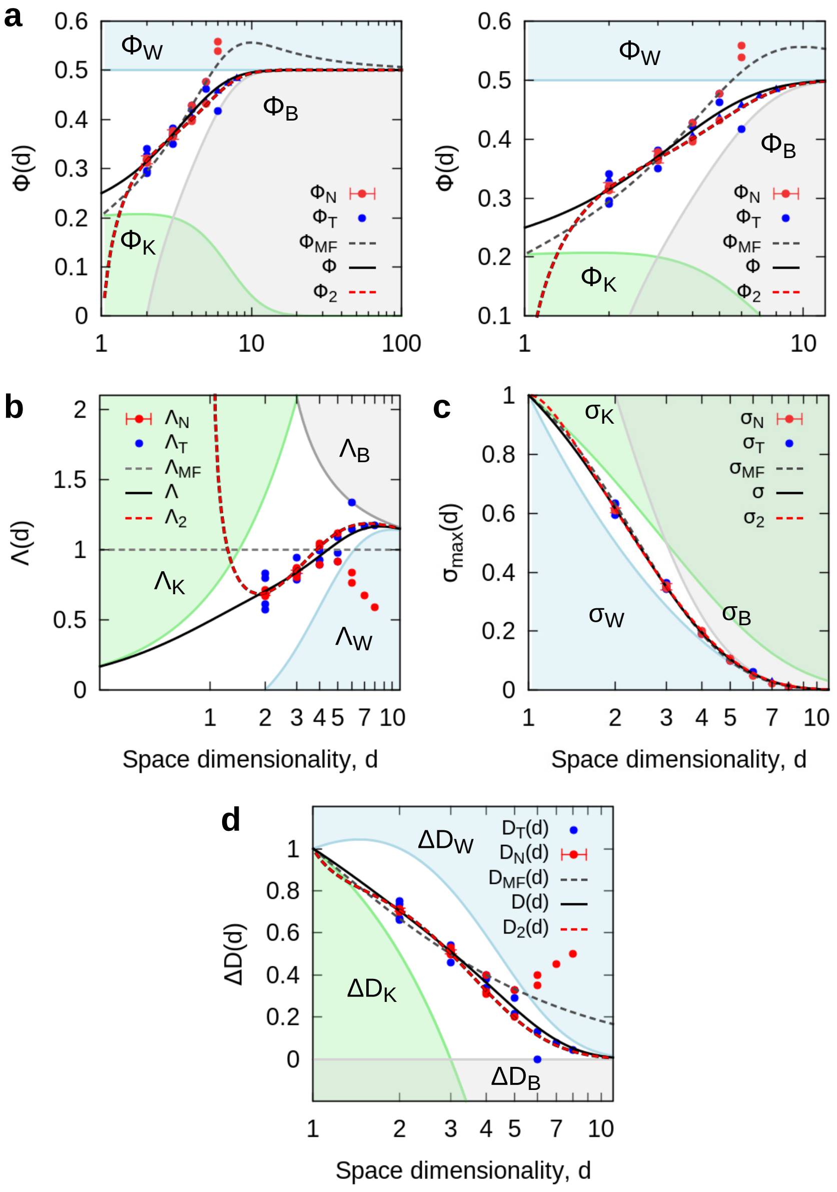

Supplementary Figure S1: Semi-log plots of (a) the effective potential $\Phi(d)$, (b) $\Lambda(d)$ function, (c) maximum-growth probability, $\sigma_{max}(d)$, and (d) DLA dimensions given as $\Delta D = D-(d-1)$. In all plots, these quantities $Y = \{\Phi, \Lambda, D, \sigma\}$ are shown as estimated through data (Y_N, Y_T), with their corresponding mean-field description (Y_{MF}), analytical solution (Y), and alternative solution (Y_2). The Kesten, Ball, and Wang boundaries are also indicated.
